# Finite element modelling of the developing infant femur using paired CT and MRI scans

**DOI:** 10.1371/journal.pone.0218268

**Published:** 2019-06-18

**Authors:** A. P. G. Castro, Z. Altai, A. C. Offiah, S. C. Shelmerdine, O. J. Arthurs, X. Li, D. Lacroix

**Affiliations:** 1 INSIGNEO Institute, Dept. of Mechanical Engineering, University of Sheffield, Sheffield, United Kingdom; 2 IDMEC, Instituto Superior Técnico, Universidade de Lisboa, Lisbon, Portugal; 3 Dept. of Oncology and Human Metabolism, University of Sheffield, Sheffield, United Kingdom; 4 Dept. of Radiology, Great Ormond Street Hospital for Children, London, United Kingdom; 5 UCL Great Ormond Street Institute for Child Health, University College London, London, United Kingdom; University of Zaragoza, SPAIN

## Abstract

Bone finite element (FE) studies based on infant post-mortem computed tomography (CT) examinations are being developed to provide quantitative information to assist the differentiation between accidental and inflicted injury, and unsuspected underlying disease. As the growing skeleton contains non-ossified cartilaginous regions at the epiphyses, which are not well characterised on CT examinations, it is difficult to evaluate the mechanical behaviour of the developing whole bone. This study made use of paired paediatric post mortem femoral CT and magnetic resonance imaging (MRI) examinations at two different stages of development (4 and 7 months) to provide anatomical and constitutive information for both hard and soft tissues. The work aimed to evaluate the effect of epiphyseal ossification on the propensity to shaft fractures in infants. The outcomes suggest that the failure load of the femoral diaphysis in the models incorporating the non-ossified epiphysis is within the range of bone-only FE models. There may however be an effect on the metaphysis. Confirmation of these findings is required in a larger cohort of children.

## Introduction

Bone fractures in the United Kingdom account for 10–25% of accidental injuries in children [1]. Of these, long bones (e.g., femur, tibia or radius) have the highest fracture rates [2–4]. A survey conducted of 382 children aged 2–14 years old found that 41.6% of fractures occurred at home [5]. It has also been reported that 25% of injuries in children aged 12 months or younger are inflicted [6,7]. Most fractures seen in child abuse occur in children younger than 3 years old, with 80% occurring before 18 months [2,4]. The determination of whether the injuries are accidental or not depends largely on clinician’s experience, as no reliable quantitative diagnostic tools are available [3,8,9]. Diagnosing child abuse continues to be a challenging task for experienced clinicians with potential negative consequences. Reports have shown that a significant number of infant child abuse cases are at first misdiagnosed (or missed) [2,10], which may lead to further harm [9,11,12]. Given this scenario, there is a need to clarify the mechanisms of childhood injury, particularly in children yet to develop appropriate communication skills [4,6].

The growing skeleton contains non-ossified cartilaginous regions at the epiphyses (up to 35% non-ossified in infants [13]) and the femoral head only completely ossifies around 14 years of age [14,15]. Therefore the femoral head is not well characterised by computed tomography (CT) scans. In contrast, magnetic resonance imaging (MRI) shows cartilage more clearly, providing anatomical information for soft tissues, while avoiding exposure to radiation [15–18]. Although finite element (FE) models available in the literature are not fully representative of the complete age range of younger children, they already provide preliminary quantitative information to differentiate accidental from inflicted injury [19–22]. However, none of these models include geometric detail of the ossifying regions of the long bone. A combination of CT and MRI would therefore lead to the development of improved FE models of immature long bones (with more precise geometries).

Previous work on paired CT/MRI examinations include evaluation of the human temporal bone [23] and the porcine femur [24]. In the specific area of FE models generated from CT and MRI, the range of applications include the human adult tibiofemoral joint [25] and the intervertebral disc [26]. However, to the authors’ best knowledge, co-registration of the two imaging modalities towards modelling of human infant bone has not been previously reported.

This work proposes a methodology to combine femoral CT and MRI examinations of the same child, exploiting the advances in commercially available imaging and modelling software. This new framework is intended to contribute to the development of more complete FE models of growing infant bones, incorporating the contribution of tissues at different levels of mineralisation [8,14,27,28]. An immediate application of this work is to elucidate the mechanisms of metaphyseal fractures [20], which is not well studied due to the lack of information or method to capture the ossifying region of the epiphysis. In the long term, such an approach is expected to enhance our understanding of the biomechanics of the developing femoral head [3,19,29,30] and differentiating accidental from inflicted injury.

## Methods

We used paired post-mortem CT and MRI scans of two infants (age 4 and 7 months), selected from the post mortem paediatric and perinatal imaging database of the Radiology Department, Great Ormond Street Hospital, London [31,32]. Cause of death was not disclosed. However, images were reviewed by experienced radiologists to ensure that the skeleton appeared normal on the scans. Ethical approval and parental consent were obtained for the use of these images for research purposes (LREC 13/LO/1494). At the time of the study, there were only two cases from this database having at least the proximal part of the femur clearly visible on both imaging modalities and aged under 18 months (4 and 7 months), and therefore still having a substantial portion of ossifying cartilage in the epiphysis.

**Fig 1:**
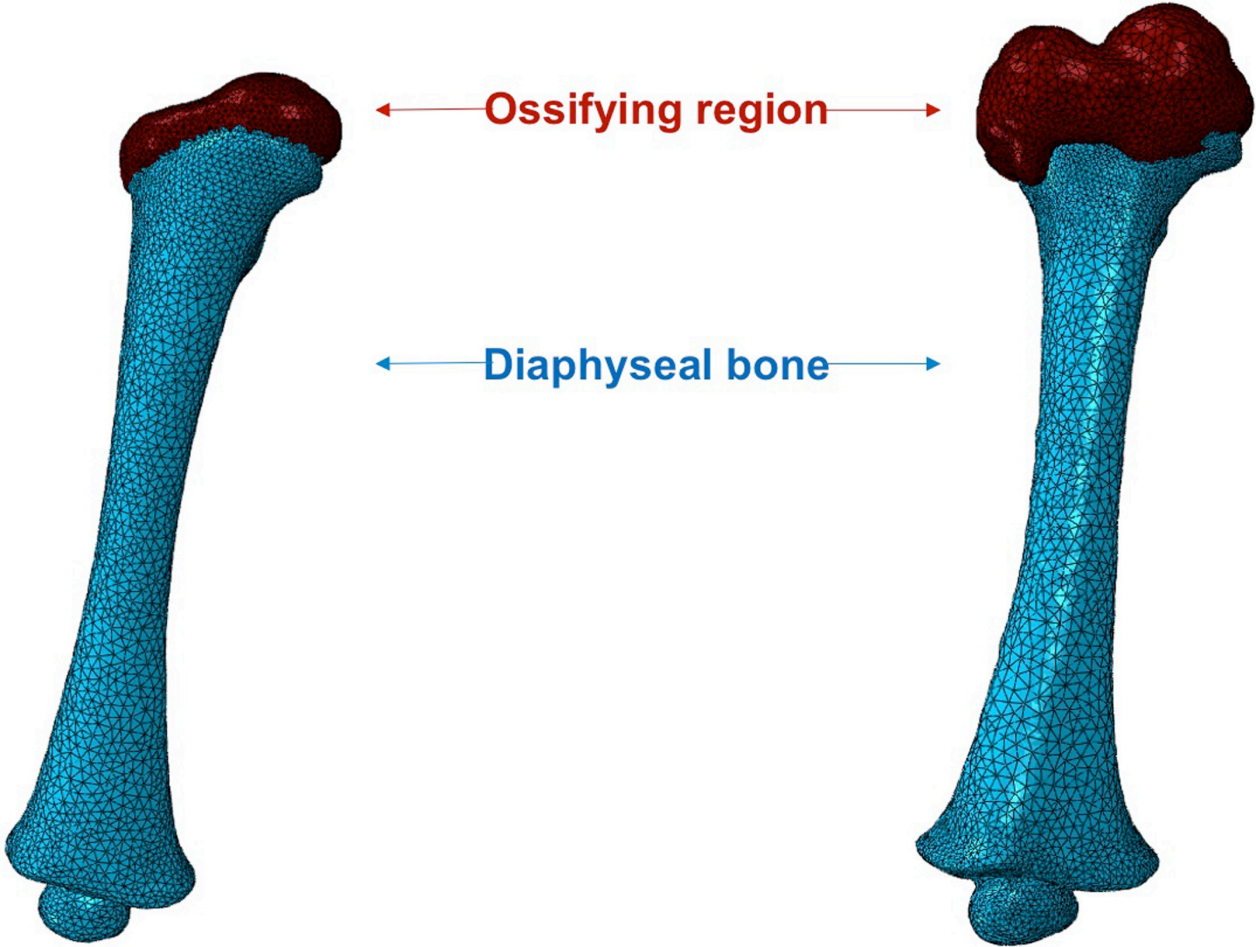
FE models of infant femur (left– 4 months; right– 7 months). Femoral dimensions are given in Table 1. Please note that only the proximal ossifying region was present in the MRI scans, i.e., the distal ossifying region was not extracted from the MRI scans.

The bony geometry of the femoral diaphysis was obtained from CT examinations and the cartilaginous tissues of the ossifying proximal femoral head region were obtained from MRI. The protocol consisted of independent segmentation of the CT and MRI data, using Amira 6.3 (FEI Visualization Sciences Group, France). CT segmentation was performed semi-automatically (through adjustable threshold), while MRI segmentation was manual. Using the multiplanar views tool available in Amira, the segmented regions of interest were matched and aligned. This alignment (and repositioning) was based on the manual alignment of the ossifying region with respect to the femoral diaphysis, using the epiphyseal area as the reference. Independent 3D surface models were then generated and imported into ScanIP 7.0 (Simpleware Ltd, UK). This software allows for the combination of the independent surface models into a single surface model, resulting in the generation of a FE model that contains both the bony diaphysis and the cartilaginous proximal femoral head (Fig 1). This protocol ensures a continuous subject-specific geometry of the femur and can be readily applied to other developing long bones.

**Fig 2:**
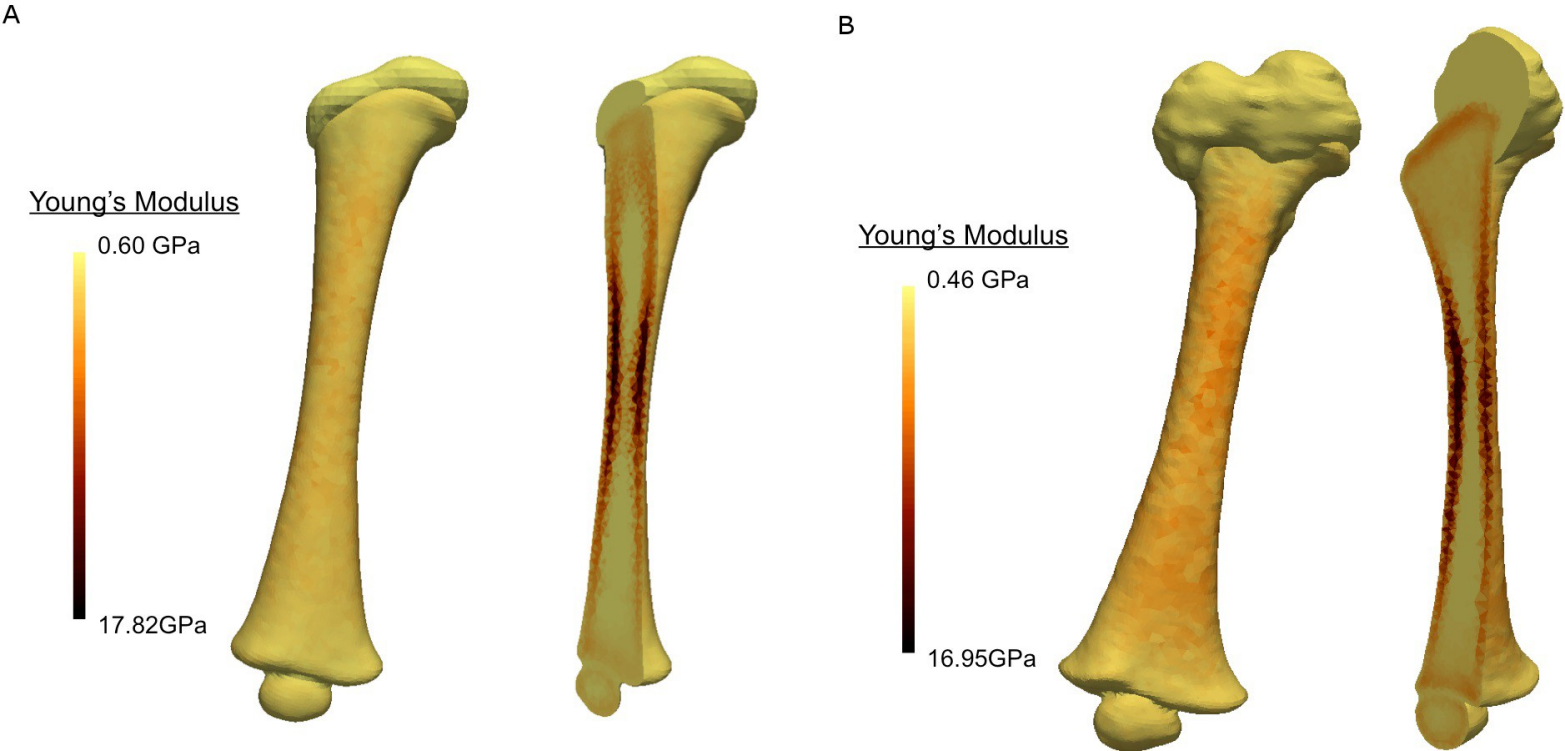
Subject-specific material properties and distribution. a) 4 months; b) 7 months. The maximum stiffness in the cortical bone is approximately 16–18 GPa for both models. The average Young’s modulus for the ossifying region is estimated to be between 0.46 and 0.60 GPa. The transition between ossifying and mineralised bone can be clearly seen towards the proximal and distal ends.

The 4-month-old model had 137493 nodes and 94922 elements, while the 7-month-old model had 161969 nodes and 111261 elements. Both models used 10-node tetrahedral elements. The material properties of each FE model were subject-specific (estimated from CT attenuation) and estimated using Bonemat V3 (Rizzoli Institute, Italy) [33]. This is in contrast to common material properties used in infant studies, which are usually scaled-down from adult data [14,34]. This software calculates an averaged Young's modulus for each element, integrated from surrounding pixels in the original CT scans [19,33,35]. The calibration methods and mathematical relationships applied were taken from Li et al. (2015), from which further information on image calibration and material property extraction from Bonemat is available. The distribution of Young's modulus along each model is shown in Fig 2, where a smooth transition between diaphyseal and ossifying regions can be seen. It must be highlighted that the properties of the ossifying region (i.e., the cartilaginous epiphysis) were also estimated from the CT scans (since this region could be considered transitional between cartilage and bone), in order to provide subject-specific material estimation for the porohyperelastic material (as described in the following paragraph).

Despite the significant difference in the size of each femur, the Young's modulus range is similar (0.60–17.82 GPa for the 4 month old infant and 0.46–16.95 GPa for 7 the month old) and aligned with the known literature [36–38]. The cross-sectional epiphyseal area between the diaphysis and the ossifying region was calculated, as well as the volume of the proximal epiphyses: the 7 months epiphysis is about 6.4 times that of the 4-month old (see Table 1). The areas corresponding to the diaphysis were modelled as isotropic linear elastic (as per Bonemat’s attribution); a detailed description of the material model can be found in Li et al. (2015). The ossifying region was modelled as porohyperelastic (linear permeability and Neo Hookean solid model), following a widely used approach to model cartilaginous soft tissue. The Neo Hookean parameters (*C*_*10*_ and *D*_*1*_, in Eq 1, which are associated with the stiffness of the material) were calculated [39] from the Young’s modulus value (E) extracted from Bonemat, using Eqs 2 and 3, where *ν* is the Poisson’s ratio, with a value of 0.20, taken from the literature [20,40]. Poroelastic properties were also extracted from the literature [41]. A summary of the material properties is shown in Table 2.

**Table 1:** Dimensions of the infant femur FE models.

	4 months	7 months
**Proximal-distal length (cm)**	10.55	13.28
**Epiphyseal cross-sectional area (cm^2^)**	2.14	3.37
**Proximal epiphysis volume (cm^3^)**	1.24	7.91
**Total volume (cm^3^)**	13.13	30.79

**Table 2:** Material properties of the ossifying region (proximal epiphyses) for the two models.

	4 months	7 months
**Permeability (m^4^/Ns)** [41]	0.00455	
**Void ratio** [41]	4.50	
**Poisson’s ratio** [20,40]	0.20	
**C_10_ (MPa)**	125.34	96.09
**D_1_**	0.0060	0.0078

$${\overset{-}{W}}_{NH}\left(C\right)=C_{10}\left({\overset{-}{I}}_{1}-3\right)+\frac{1}{D_{1}}{\left(J-1\right)}^{2}$$ (1)

$$C_{10}=\frac{E}{4\left(1+\nu \right)}$$ (2)

$$D_{1}=\frac{6\left(1-2\nu \right)}{E}$$ (3)

**Fig 3:**
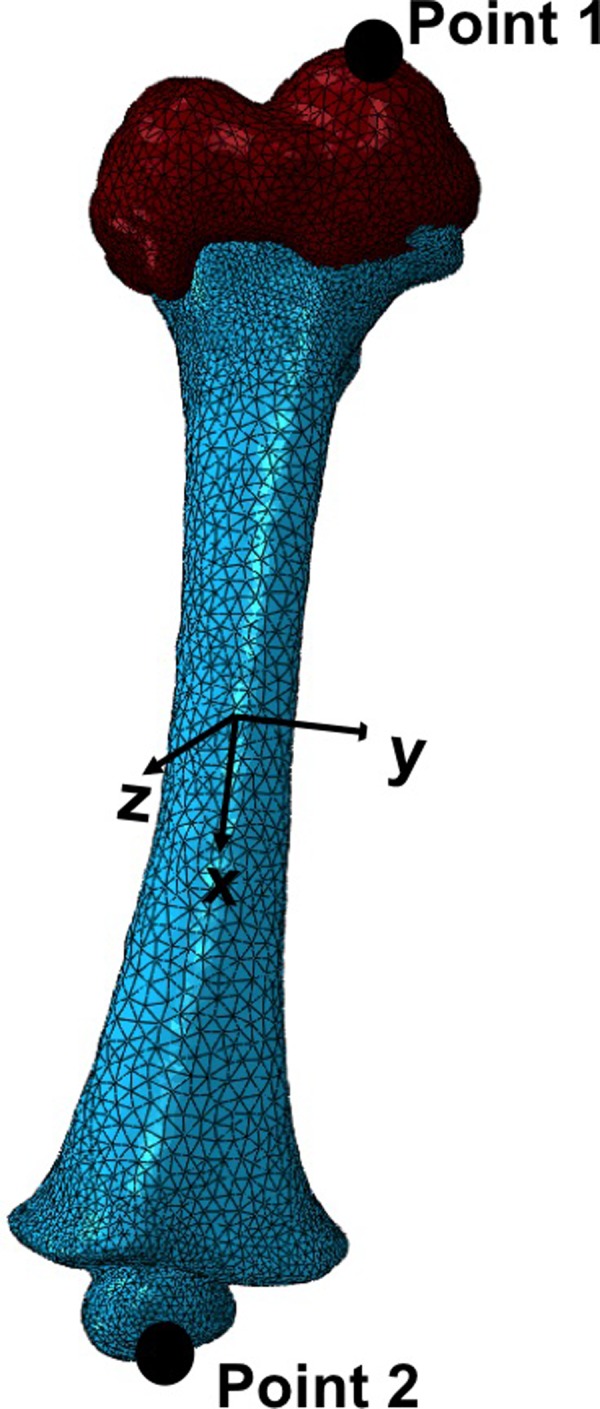
Coordinates system for the infant femur, with the reference points for applying the loading conditions.

In order to obtain more accurate boundary conditions, previous work proposed a coordinate system for the femur where two cross-sections of the diaphysis were identified at 25% and 75% of its total length [22]. The same coordinate system was used in this study in order to compare current results with those of the previous publication. The centroids of these two cross-sections were estimated, and the X-axis was defined as a line passing through both centroids, running from proximal to distal. A positive Y-axis points medially and a positive Z-axis points to the anterior perpendicular to the X-Y plane. This coordinate system (Fig 3) ensures the minimisation of the implicit bending effect due to anatomical asymmetry of the femur [19,22]. The anatomical reference points (1 and 2) were also selected to be consistent with the bone only study by Li et al., 2015 [19], in which inter- and intra-observer reliability tests revealed these points to be the most reliable landmarks.

Two sets of experiments were carried out, namely torsion and uniaxial loading. Both loads were applied at Point 1, keeping the femur fixed for all degrees of freedom at Point 2. Torsion around the longitudinal axis was applied to mimic a twist to the femur, and the three uniaxial loads were chosen to represent direct impact load applied to the bone (at Point 1) in each of the X, Y, and Z directions. Point 1 was selected for application of the load because it is the ossification centre for the proximal femur, which will become the femoral head once fully mineralised. This is in line with the approach taken in studies of adult femurs.

For torsion, external moment of 2kN.mm was applied, in order to compare the moment to fail of these models (femur with bone plus ossifying region) with the analogous simulations performed by Altai et al. (2018) [22] in their model (femoral diaphysis only). The moment to fail corresponds to the maximum strains or the threshold of elastic strain limit, which were reported to be 0.73% in tension and 1.04% in compression, following the works of Bayraktar et al. (2004) [42] and Schileo et al. (2007) [43] on adult bone. It must be highlighted that only the diaphysis was considered for this calculation. For the uniaxial loads of 200N, different directions were selected to reveal which efforts would be more relevant to induce highly localised stress-strain levels on the infant femur and potentially lead to metaphyseal or shaft fractures. The FE simulations were performed with Abaqus 6.13 (Dassault Systèmes Simulia Corp., USA).

## Results

**Fig 4:**
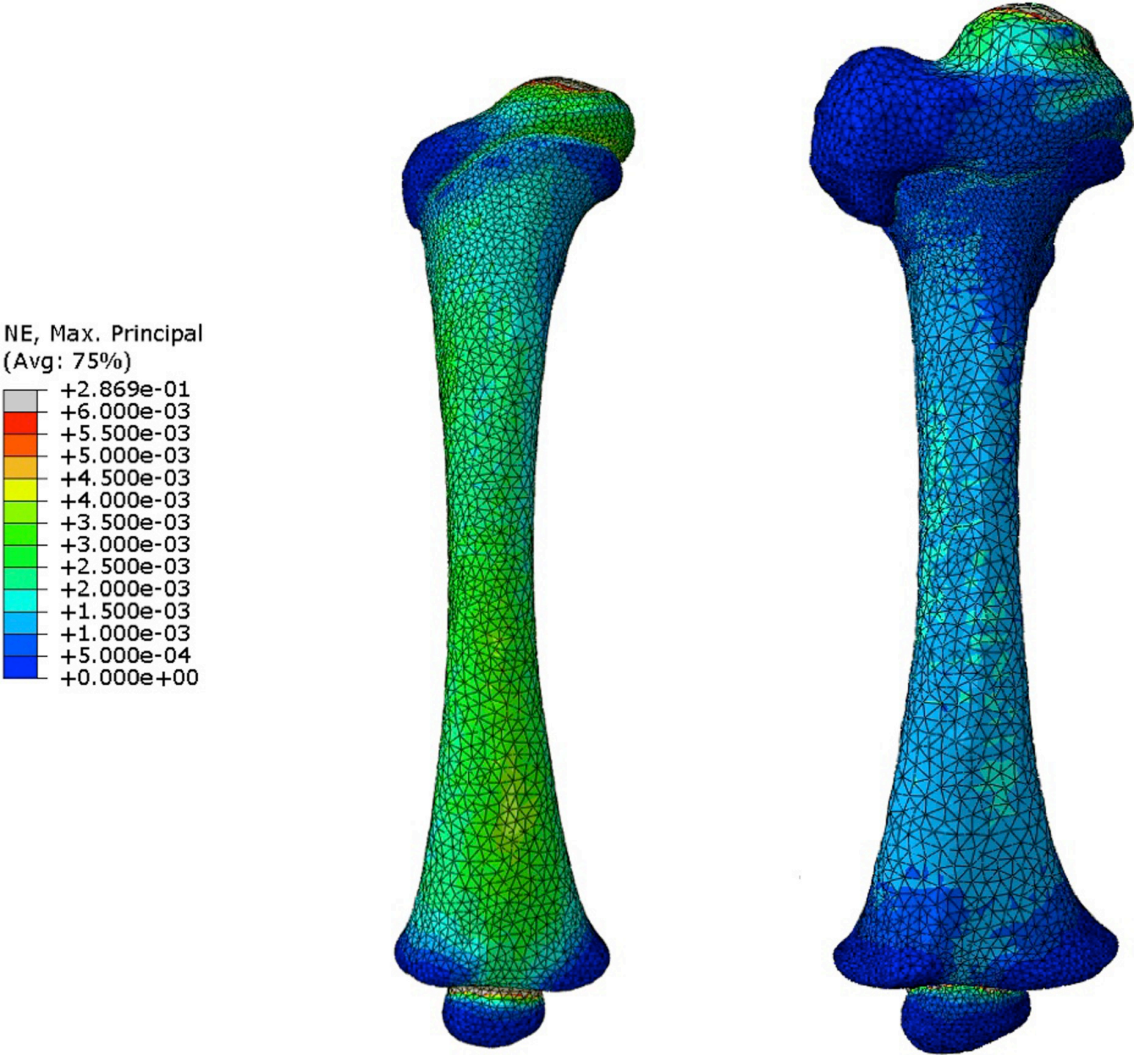
Maximum principal strain distributions for a 2kN.mm external moment applied on infant femur (left– 4 months; right– 7 months).

**Fig 5:**
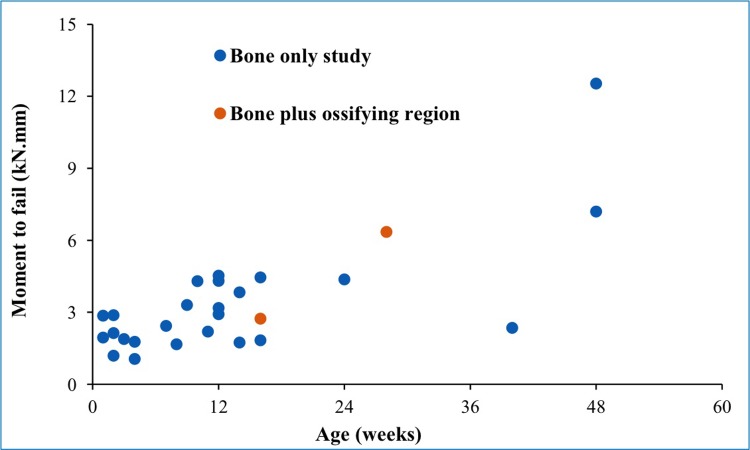
Moment to fail of infant femurs, comparing bone only calculations from Altai et al. (2018) with current results for bone plus ossifying cartilaginous region. Note that a different CT-only dataset was used in Altai et al. (2018).

The maximum principal strain distributions in the two femur models under torsion are shown in Fig 4. The maximum principal strain range on the 4 months model was 4.1x10^-06^ to 2.9x10^-01^, compared to 9.0x10^-07^ to 5.3x10^-02^ on the 7 months model. The area of high strain was located on the femoral diaphysis. The developing greater trochanter appeared to be almost strain-free, while some strain concentration appeared to build up around the epiphyses, particularly in the 4 months model. The moment to failure evaluated in these two models were compared against the values obtained from previous simulations performed exclusively on the femoral diaphysis [22], which yielded very similar results. The comparison of moment to fail calculations is shown in Fig 5.

**Fig 6:**
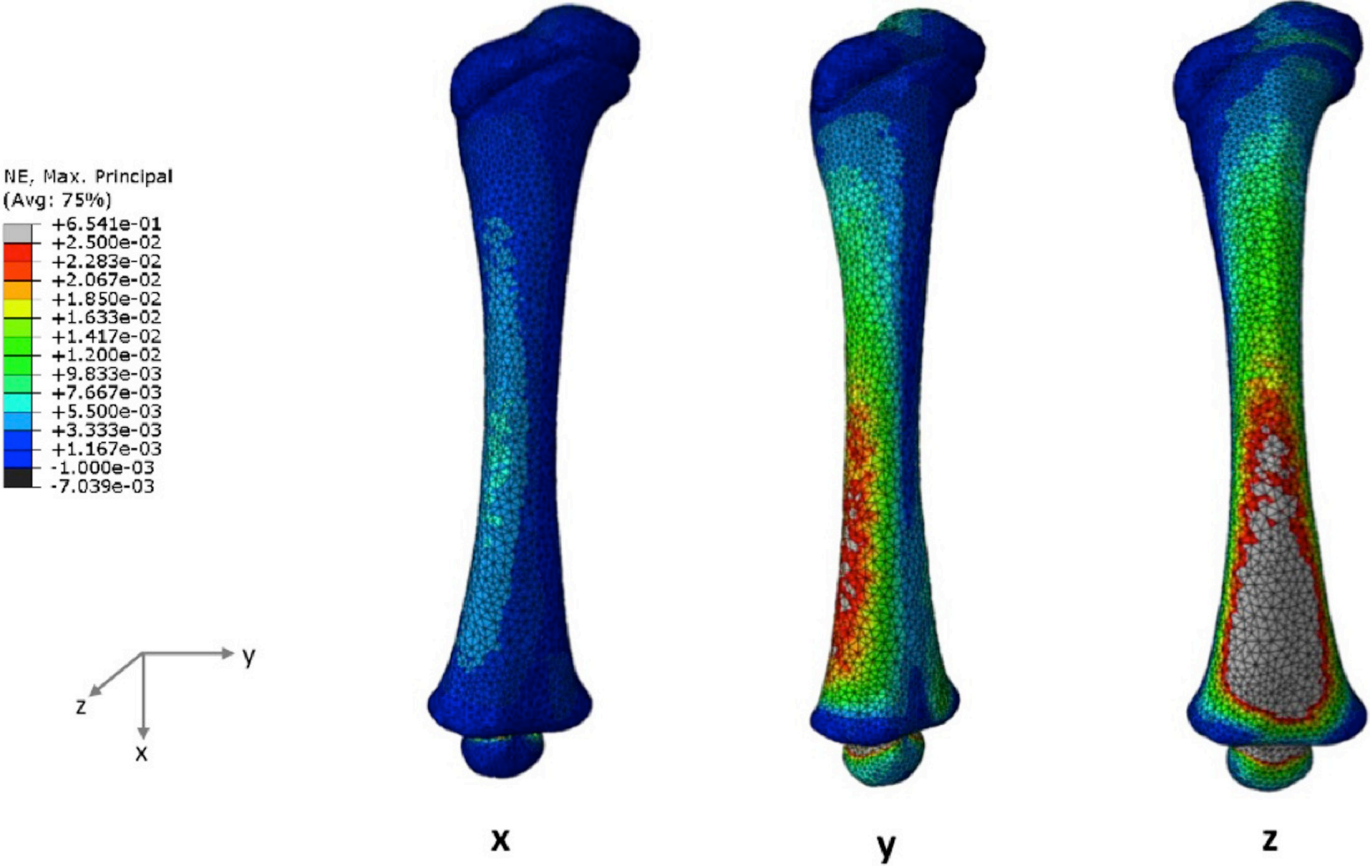
Maximum principal strain distributions when a uniaxial load is applied in the X-, Y- or Z-axis for the 4 months model.

**Fig 7:**
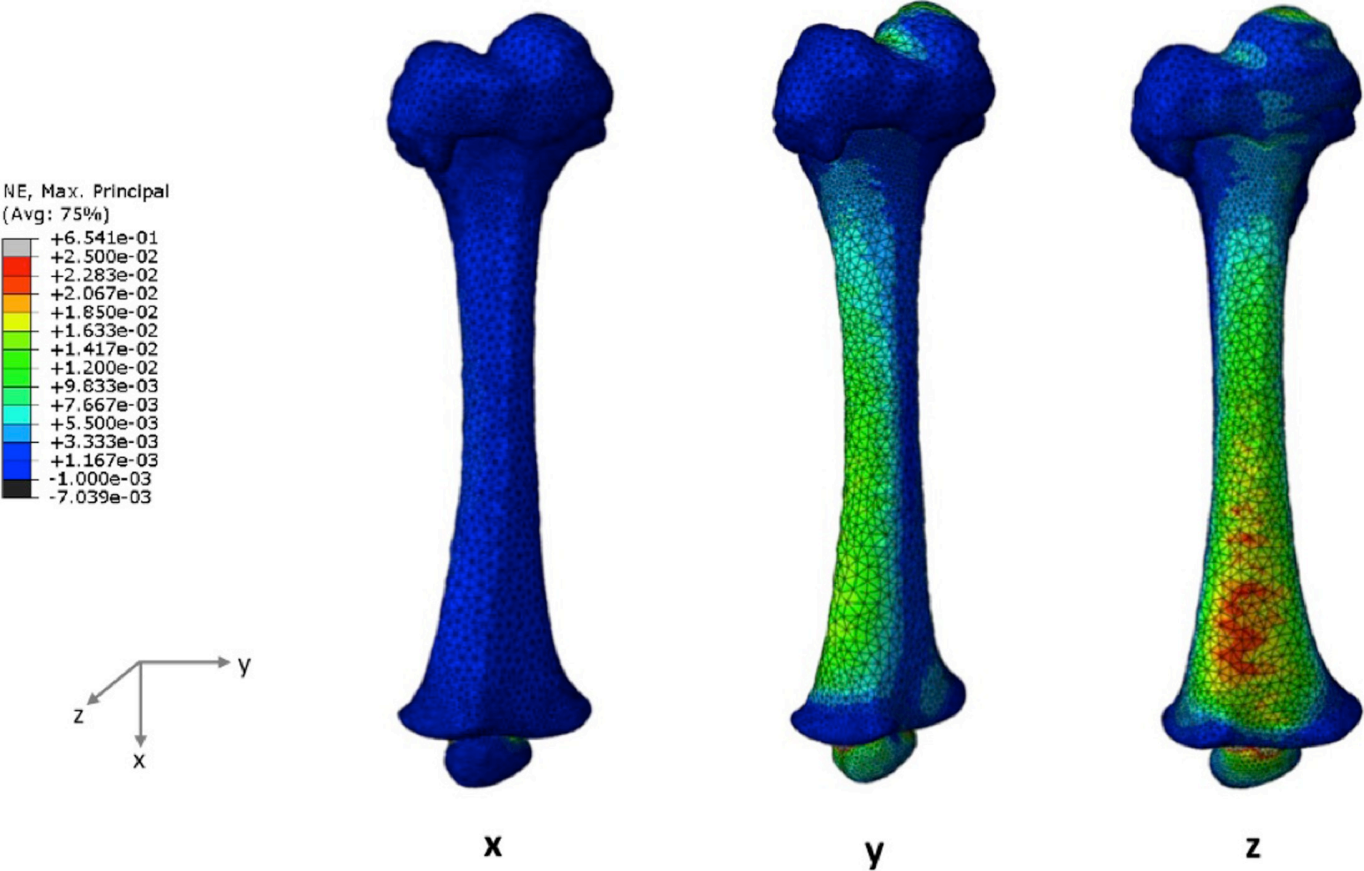
Maximum principal strain distributions when a uniaxial load is applied in the X-, Y- or Z-axis for the 7 months model.

The maximum principal strains under uniaxial loads predicted for the 4 months model are visibly higher than those of the 7 months model, as shown in Fig 6 (4-month-old) and Fig 7 (7-month-old), respectively. Maximum principal strains is plotted since the bone first failed in tension.

These uniaxial loads seem to be promoting strain accumulation on the diaphyseal regions, which means the epiphyses were less deformed. Femoral head deformation was most evident following the bending effect of the load applied on the Z-axis (anterior-posterior direction), regardless of developmental stage of the femur.

## Discussion

This study suggests that the proximal epiphysis is not as significant for the biomechanics of the whole infant femur as has previously been speculated [20,44,45]. The comparison with FE models of bone only infant femora models [22] showed an agreement with the moment to fail, for both the 4 and the 7 months old subjects.

Regarding the characterization of the proximal ossifying region, it was observed that the material transition from the diaphysis to the cartilage-based soft tissues is smooth, reflecting the mineralisation process. Although the method for calibration applied through Bonemat was originally developed for bony material on CT scans, the authors believe this is the best possible approach to obtain representative mechanical properties of that individual given the scarce data available to describe children’s cartilage material properties. An alternative approach would be to assume adult articular or hyaline cartilage property for this region, which is likely to differ from infants. The thickness of the ossifying region is very different from regular cartilage layers, and the current knowledge of the mineralisation process of developing bone suggests that this region is formed of an intermediate material between bone and cartilage [8,18,27,29]. Plus, the material properties extracted from Bonemat (Young Modulus in the range of 460–600 MPa) indicate towards a transitional material between what is known for adult cartilage (Young Modulus in the range of 1-10MPa [46,47]) and bone. Another limitation of this study is that the bone was modelled as isotropic linear elastic. Although this is a common simplification, the effect of anisotropy on the whole bone (including ossifying region) needs to be addressed in the future.

Pure uniaxial compression through the proximal-distal length of the femur could not be achieved due to asymmetry of the femur. These loading conditions resulted in a combination of lateral compression and bending. As such, the effect of the direct load (200N) was reduced, particularly when compared with the bending caused by the other two uniaxial loading conditions (Y- and Z-axes in this coordinate system). These conditions help to extrapolate how the infant femur responds to potentially harmful pushing or pulling movements in different directions. Given that the major deformation occurred with frontal bending loads (Z-axis) and that this movement is likely to be clinically associated with inflicted injury, one can speculate that frontal plane infant femoral injuries/fractures have greater probability of being associated with physical abuse through excessive loading than torsion or other uniaxial loads [7,29,45]. It should be also noted that although the loading conditions applied here were simplified, as in other work on developing bones (e.g., Tsai et al. 2017), they were related to the magnitude and direction of abuse-inflicted injuries. Typically, a combination of these loads would occur in a real-world infant injury scenario, such as those causing the classic metaphyseal lesion [48]. To the authors’ best knowledge, there has been only one previous modelling paper on the classic metaphyseal lesion [20], in which the authors excluded the epiphysis, making the assumption that “any influence of the physis or ligaments on the relative strain patterns would be negligible”. However, the current study does indicate that the strain distribution changes within the transition region and this is worth further investigation. The biggest impacts of including the epiphysis seems to be twofold: (a) there is now an area of concentrated strain at the transition region; and (b) the epiphysis itself suffers from highly localised strain, even though the overall mechanical behaviour of the shaft is largely unaffected. Our results indicate that injuries close to the epiphysis should be modelled with caution, since the mineralising cartilaginous epiphysis does seem to have an effect on local strain distribution.

There is very limited paired CT/MRI imaging data available containing both the femoral diaphysis and proximal femoral head, contributing to the lack of previous studies on the non-ossified regions of the long bones, but the two subjects in this current study show proof of principle in the target population. This would provide a new and non-invasive image-based approach to investigate young children’s bone properties, complementing previous mechanical studies of cadaveric bone samples, which are also scarce [10,36–38]. The developed approach could also be useful to validate failure predictions on paired CT-MRI FE models of young animal bones, by comparing with destructive results. Future work should include more infant cases considering a wider range of developmental stages, up to 3 years old [4]. The establishment of well-defined landmarks or accurate measurement and marking of the epiphyseal area may improve the accuracy of the process, although manual adjustments might still be needed to ensure consistent representation of the femoral anatomy.

In conclusion, this work introduces a new approach to incorporate both bone and ossifying cartilage in FE models of the infant femur, on both geometrical and constitutive aspects of modelling. The results suggest that the proximal ossifying region has no significant effect on the moment to fail of the infant femoral shaft under torsion, although axial loading in the proximal femur produced variable results around the metaphyseal region that require further investigation.
